# National survey of enhanced recovery after thoracic surgery practice in the United Kingdom and Ireland

**DOI:** 10.1186/s13019-020-01121-2

**Published:** 2020-05-14

**Authors:** Alina-Maria Budacan, Rana Mehdi, Amy Pamela Kerr, Salma Bibi Kadiri, Timothy J. P. Batchelor, Babu Naidu

**Affiliations:** 1 Department of Thoracic Surgery, Heartlands Hospital, Bordesley Green East, Birmingham, B9 5SS UK; 2 Department of Thoracic Surgery, University Hospitals Bristol NHS Foundation Trust, Upper Maudlin Street, Bristol, BS2 8HW UK; 3 Institute of Inflammation and Ageing, College of Medical and Dental Sciences, Centre for Translational Inflammation Research, University of Birmingham Laboratories, Queen Elizabeth Hospital Birmingham, Edgbaston, Birmingham, B15 2TT UK

**Keywords:** ERAS, Enhanced recovery, Thoracic surgery, Lobectomy

## Abstract

**Background:**

Evidence that Enhanced Recovery After Thoracic Surgery (ERAS) improves clinical outcomes is growing. Following the recent publications of the international ERAS guidelines in Thoracic surgery, the aim of this audit was to capture variation and perceived difficulties to ERAS implementation, thus helping its development at a national level.

**Methods:**

We designed an anonymous online survey and distributed it via email to all 36 centres that perform lung lobectomy surgery in the UK and Ireland. It included 38 closed, open and multiple-choice questions on the core elements of ERAS and took an average of 10 min to complete.

**Results:**

Eighty-two healthcare professionals from 34 out of 36 centres completed the survey; majority were completed by consultant thoracic surgeons (57%). Smoking cessation support varied and only 37% of individuals implemented the recommended period for fluid fasting; 59% screen patients for malnutrition and 60% do not give preoperative carbohydrate loading. The compliance with nerve sparing techniques when a thoracotomy is performed was poor (22%). 66% of respondents apply suction on intercostal drains and although 91% refer all lobectomies for physiotherapeutic assessment, the physiotherapy adjuncts varied across centres. Perceived barriers to implementation were staffing levels, lack of teamwork/consistency, limited resources over weekend and the reduced access to smoking cessation services.

**Conclusion:**

Centres across the UK are working to develop the ERAS pathway. This survey aids this process by providing insight into “real life” ERAS, increasing exposure of staff to the ESTS- ERAS recommendations and identifying barriers to implementation.

## Background

The ERAS concept pioneered by Kehlet in the 1990s aims to minimise surgical stress following elective surgery [1]. Adopting a standardized evidence based protocol has been shown to improve clinical outcomes in general surgery [2, 3]. In thoracic surgery, the publication of the ERAS society and the European Society of Thoracic Surgeons recommendations for enhanced recovery after lung surgery [4] identifies forty five core elements, which cover the peri-operative period, from pre-admission to discharge. Given the diversity of practice in thoracic surgery and the fact that operations are most commonly performed for lung cancer [5], these guidelines focus on patients undergoing lobectomy. Despite the growing body of evidence, challenging deeply rooted perioperative practices requires a culture shift from traditional surgical and anaesthetic dogma. Healthcare professionals (HCPs) beliefs can hinder the implementation of such a programme, if they are not familiar with the ERAS principles and do not perceive its benefits [6]. Importantly it is incorporated into the Society of Thoracic Surgeons Patients’ Guide to Heart, Lung and Oesophageal Surgery [7].

Understanding the situation from a country-wide perspective in terms of current delivery of the core elements, and healthcare practitioners knowledge and beliefs around them is the first step to developing an international strategy to successfully implement an ERAS programme in thoracic surgery [8]. Potentially there could also be major logistic issues that could hamper delivery therefore identifying these will be important too.

Thus, the aim of this national audit was to capture variation in enhanced recovery pathway practices and identify perceived barriers to implementation in patients undergoing lobectomy for lung cancer in thoracic surgery centres across the UK and Ireland, through a web-based survey.

## Material and methods

### Study design and participants

A national multicentre audit was conducted to assess the knowledge and current practice of the ERAS pathway for patients undergoing lung lobectomy surgery across the UK. We performed a retrospective analysis of prospectively collected data. As this project was carried out as an audit into current ERAS practice, intended to measure practice of the participating units, following internal review and after using the NHS Research Ethics committee approval tool [9], we decided an IRB was not required.

The anonymous survey was developed using the method described by Artino et all [10] and comprised of 38 open, closed and multiple-choice questions, which evaluated the main domains of ERAS in the peri-operative period; additional information regarding the most and least successful aspects of the pathway was recorded. Some questions had an open-ended response; these were collated and categorised based on similar themes then supplemented with quotes to add detail to the point being made. Descriptive coding was used to gain further insight into how the pathway is implemented at various centres.

The web-based survey hosted at SurveyMonkey™ (www.surveymonkey.com, Portland, Oregon, USA) was distributed electronically via email to all practising cardiothoracic surgeons and nurses listed in the Society for Cardiothoracic Surgery in Great Britain and Ireland and National Lung Cancer Forum for Nurses databases across the 36 centres that perform lung lobectomy surgery in the UK on the 23rd of July 2018. The mailing list contained 962 email addresses. The strategy of a much wider call which included professionals who are not directly involved in the care of the thoracic surgical patient (i.e. palliative care, cardiac surgery) was taken to ensure we do not miss any HCPs who are directly involved. The following professional groups participated: thoracic surgeons (trainees and consultants), nurses involved in the care of the thoracic surgical patient (including lung cancer nurse specialists and thoracic advanced clinical practitioners) and physiotherapists.

The questionnaire took an average of 10 min to complete and three reminder emails were sent every 30 days to encourage involvement. If a unit did not respond, reminders were sent until an increase in response rate was achieved. The survey period closed on the 15th of May 2019.

### Statistical analysis

The results were downloaded and reviewed by members of the research team. Descriptive statistics and the filter tools inherent in the SurveyMonkey™ software were used to compile, sort and present the categorical variables as percentage. The continuous variables were reported as median and interquartile range using the Microsoft® Office Excel 2016 software.

## Results

### Sample characteristics

Eighty-two valid responses from HCPs across 34 out of the 36 centres (response rate 94%) that perform lung lobectomy surgery in the UK were analysed. Though a total of 125 responses were received, 3 worked in centres that do not perform lobectomy and 40 answered only the first question and so were excluded. Responders were mainly consultant cardiothoracic surgeons (57%) followed by senior nurses (24%), registrars (9%), physiotherapists (5%) and other members of staff (5%) involved in delivering the ERAS pathway.

**Fig. 1:**
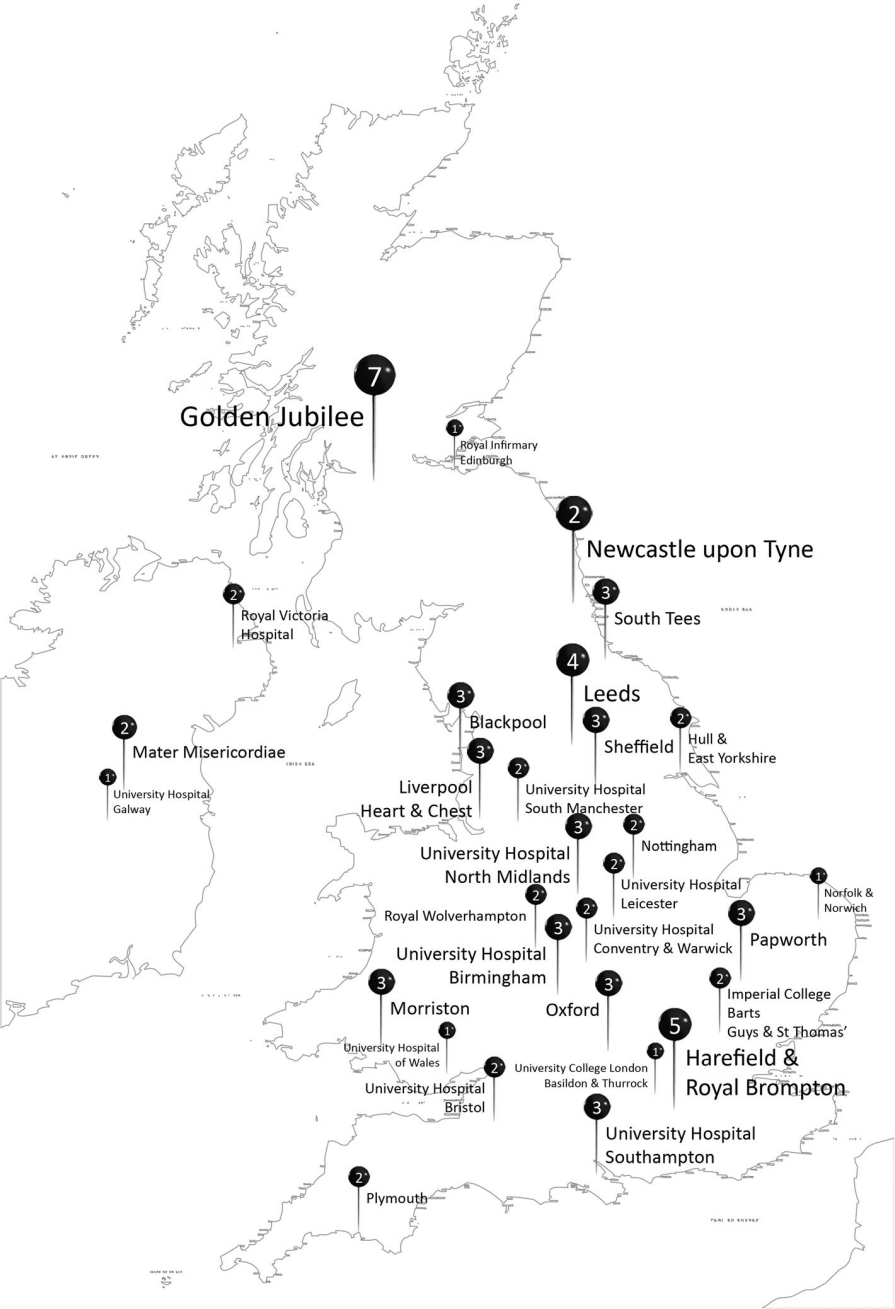
Map demonstrating the participating centres and the number of responses per unit

A map with the participating centres and the number of responses per unit can be found in Fig. 1.

### Pre-operative ERAS elements

The preadmission information, education and counselling was adequate, although the methods used across centres varied, with only a small percentage having the possibility to provide a DVD or website. Pulmonary rehabilitation was offered by 65% of respondents and smoking cessation support varied across centres. Six percent of participants reported they do not offer any routine support and 5% were unsure. Those unsure were thoracic surgeons at various career stages (senior house officer, registrar and consultant) from different units.

Perception of the abstinence period prior to surgery varied; 46% considered less than 4 weeks is enough to observe a clinical benefit (Table 1).

**Table 1:** Smoking cessation

***Support (n = 82)***	
Nicotine replacement therapy prescribed in secondary care	51%
Referral to hospital-based smoking cessation services	54%
Referral to general practitioner (GP)	37%
Referral directly to community smoking cessation services	39%
No routine support offered	6%
Not sure	5%
Other^a^	13%
***Abstinence timeframe (n = 82)***	
< 2 weeks	18%
> 2 weeks to < 4 weeks	28%
> 4 weeks to < 6 weeks	16%
> 6 weeks	20%
Unsure	18%

^a^smoking cessation supervised by clinician, signpost to community smoking cessation, basic advice from specialist nurse

### Admission and perioperative ERAS elements

Forty-eight respondents (59%) said they screen patients for nutritional status pre-operatively and the support for patients identified as malnourished or at risk varied and discrepancies within the same unit were noted. For example, in one centre some reported they only refer to dietician, whilst others also offer dietary advice or prescription for supplements; in another centre, one healthcare professional stated they refer to dietician whilst another did not offer any support (Table 2).

**Table 2:** Preoperative nutrition, fasting and carbohydrate treatment

**Support for patients identified as malnourished or at risk (*n*** **= 82)**	
Referral to dietician	66%
Dietary advice/ prescription for dietary supplements	51%
Referral to GP	33%
Other^a^	4%
Not offered	6%
**Fasting (*n*** **= 82)**	
***Fluids***	
2 h	37%
> 2 to < 4 h	21%
>4 to <6 h	21%
> 6 h	15%
Other^b^	6%
***Solids***	
< 3 h	1%
> 3 to < 6 h	6%
6 h	59%
> 6 h	33%
Unsure	1%

^a^in hospital dietician, advise visit GP

^b^depends on the surgeon/anaesthetist

Whilst for solids a fasting period of 6 h or more was reported (92%), only 37% of participants implemented a fluid fasting period of 2 h. The open-ended responses highlighted the variation in fluid fasting practice. In one centre this was determined by the surgeon whilst in another the decision was made by the anaesthetist. In most cases, patients were not given preoperative carbohydrate loading (60%) (Table 2).

Forty-one participants (50%) reported that < 5% of patients undergoing lobectomy through thoracotomy received a thoracic epidural in their unit. The preferred post-operative analgesic agents were paracetamol (98%), strong opiates (91%) and weak opiates (76%). Only 55% of participants reported their patients receive NSAIDs for post-operative pain (Table 3). A small percentage reported they perform a muscle sparing thoracotomy (41%) or use intercostal nerve and muscle sparing techniques (22%). When analysing the open-ended responses, it became clear that the techniques used to reduce post-operative pain were left to surgeon preference and some spare solely the serratus anterior muscle whilst dividing latissimus (Table 4).

**Table 3:** Regional anaesthesia and pain relief

***Use of epidural in thoracotomy patients (n = 82)***	
< 5%	50%
6–25%	10%
26–75%	12%
76–100%	18%
Unsure	10%
***Postoperative analgesic options (n = 80)***^*a*^	
Paracetamol	98%
NSAIDs	55%
Weak opiates (e.g. codeine)	76%
Strong opiates (e.g. morphine)	91%
Neuropathic agents (e.g. gabapentin)	51%
Local anaesthetics agents (e.g. lidocaine patches/injections)	46%
NMDA antagonists (e.g. ketamine)	8%

*NMDA* N-Methyl-D-aspartate, *NSAIDs* Non-Steroidal Anti-Inflammatory Drugs

^a^ Postoperative analgesic used in all lobectomies (VATs and open approach)

**Table 4:** Surgical technique

***Percentage of VATS lobectomies (n = 81)***	
< 25%	2%
26–50%	19%
51–75%	33%
76–100%	38%
Not sure	8%
***Routine thoracotomy techniques (n = 80)***	
Muscle sparing	41%
Intercostal nerve sparing	22%
Not applicable	24%
Other^a^	13%

*VATS* Video-Assisted Thoracoscopic Surgery

^a^serratus sparing, surgeon preference

### Postoperative ERAS elements (Table 5)

Sixty six percent of participants reported they place the intercostal drain on suction. The cut off value for pleural fluid drainage accepted for removal of chest drain varied. Sixty four (83%) participants reported a cut off (median 300 ml, range 50-1000 ml) whilst the remaining said they do not have a specific value and base the decision to remove the intercostal drain on the type of fluid (no frank blood/chyle) and the absence of an air leak. Patients were assessed by a physiotherapist post-operatively in most centres. Physiotherapy adjuncts/strategies were available widely to aid the management of patients following lobectomy including early mobilisation within 6 h of surgery (77%), incentive spirometry (74%) and prophylactic mini tracheostomy (43%). Two centres reported inability to implement early mobilisation due to staffing level and in another centre patients used a bike to improve post-operative mobilisation.

**Table 5:** Postoperative ERAS elements

**Chest drain management- postoperative value of suction (*n*** **= 80)**	
0 kPa	26%
-0.5 kPa	8%
-1 kPa	5%
-1.5 kPa	2%
-2 kPa	38%
≥ −2.5 kPa	21%
**Early mobilisation and adjuncts to physiotherapy**	
*Routine post-operative assessment by physiotherapist (n = 80)*	
All lobectomy patients	91%
Patients undergoing a lobectomy via thoracotomy	5%
Only ‘high risk’ patients	3%
Not routinely assessed	1%
*Available physiotherapy adjuncts (n = 81)*	
Incentive spirometry	74%
Early mobilisation within 6 h of surgery	77%
Prophylactic mini-tracheostomy	43%
Non- invasive positive pressure ventilation	58%
Not sure	4%

*kPa* Kilopascal

### Perceived most and least successful aspects of ERAS

The perceived most successful aspects of ERAS were: preadmission information/counselling (74%), chest drain management (73%), VTE prophylaxis (67%), physiotherapy (65%) and surgical technique/incision (60%). Smoking cessation (44%), carbohydrate loading (45%), alcohol cessation (40%) and preoperative pulmonary rehabilitation programs (36%) were the least successful aspects of ERAS.

### Additional comments

The final question of the survey was open-ended and provided a space for additional comments on the ERAS pathway. Responses, as they pertained to different aspects of the pathway included: different practices in surgical technique, drain management and perioperative analgesia within the same unit; barriers and enablers to implementation and suggestions for improvement. These are summarized in Table 6 with examples of related comments.

**Table 6:** Themes which arouse from the open-ended questions with examples of related comments from participants

**Theme**	**Comments**
**Variation in practice within the same unit**	“Some answers may differ in individuals in the unit eg intercostal nerve sparing.” “We have 2 surgeons with completely different pathways for pain control” “Cut off value for pleural fluid drainage accepted for removal of chest drain in the first 24 h differs per consultant”
**Barriers to ERAS implementation**	“We have a quick turn around for surgery and do not currently have time to implement pre-hab.” “The biggest barrier to implementing the physiotherapy part of our ERAS is physiotherapy staffing and provision.” “We have struggled to convince our physiotherapists of the benefits of an aggressive post-operative mobilization plan or to attend pre-admission clinic.” “The greatest issues are teamwork, consistency, reinforcing the same information & having the active support of consultants & decision-making managers - rather than in word only.”
**Enablers to ERAS implementation**	“We phone follow up patients 24 h and 72 h post op. Really good support to pts. and rels ensures point of hospital contact and prevents readmissions.” “Patient education and pre-habilitation has significant role in better outcome and ERAS.”
**Suggestions for improvement**	“Moving forward, we need more resources at weekends- physio and occupational therapy especially but also pharmacy discharge team etc- we still see a weekend effect on length of stay. Also disappointing to see declining access to smoking cessation nationally- lung cancer surgery definitely a “teachable moment”.’

## Discussion

This is the first national survey that analyses the variation in practice and the perceived difficulties to ERAS implementation within thoracic surgery centres across the UK. Our results show that, despite being variably implemented, the fast track surgery principles have been widely adopted at a national level. Many of the recommendations in the ESTS- ERAS guidelines had low levels of evidence, but received a strong recommendation based on the concept that they were likely not harmful. Understanding and accepting the data used to make these recommendations and implementation in clinical practice requires years, thus the variability amongst centres is not surprising.

Our response rate is similar to that reported in a comparable survey of ERAS practice in general surgery [11]. In 6 centres we received only one response which may lead to a degree of bias but there was good concordance between multiple responses from the same unit suggesting that any misrepresentation is likely to be minimal.

### Preoperative ERAS elements

Clinicians recognise the importance of pre-operative counselling and education, therefore they provide a combination of written and oral information to their patients, as recommended by ERAS guidelines [4]. Patients want to know more about their diagnosis, recovery and coping with issues following discharge [12] and preoperative counselling has been shown to reduce stress and anxiety and set patients and carers expectations about surgery [13].

As the biggest independent risk factor for developing a post-operative pulmonary complication [14], guidelines recommend stopping smoking at least 4 weeks before the operation. Since the optimal abstinence timeframe required to observe benefits is still being debated, it is not surprising that only 36% of our respondents follow the recommendation. A survey from the United States yielded similar results, as most thoracic surgeons said they wait 2–4 weeks after smoking cessation before performing surgery. Furthermore, 47% of respondents would not perform major lung resections in patients who are current smokers [15]. In the context of the National Health System (NHS) the time constraints associated with cancer surgery have to be considered, as surgery cannot be delayed to allow patients at least 4 weeks of smoking cessation.

Two thirds of individuals comply with ERAS guidance [4] and refer patients for pulmonary rehabilitation before surgery. Reasons for not offering prehabilitation were the lack of service availability and a personal belief that it is not beneficial. This personal belief can be counter-intuitive, as thoracic surgeons use the exercise capacity as an element to determine a patients’ fitness for surgery. The risk factors for postoperative complications have a higher prevalence amongst the lung cancer patients and prehabilitation can be used to modify most of them, thus improving outcomes [16].

### Admission and perioperative ERAS elements

ERAS guidelines recommend optimising nutrition in the perioperative period, avoiding long periods of pre-operative fasting and administering carbohydrate loading drinks on the morning before surgery [4]. Historically patients have been kept nil by mouth from midnight, so the poor compliance with the fluid fasting timeframe showed by our results has a potential explanation. Qualitative data analysis provides clarification, showing that the fasting periods are decided either by the surgeon or the anaesthetist, hence the variety in clinical practice. Similar findings have been described in a 2016 ERAS survey amongst general surgeons, with only 22% of participants giving patients carbohydrate drinks on the morning before surgery and 77% reporting they fast patients for both solids and liquids from midnight [17]. These results emphasise how HCPs beliefs and knowledge can hinder ERAS implementation.

The guidelines recommend a VATS approach in early lung cancer and emphasise that paravertebral block (PVB) yields a lower risk of complications and is equivalent to thoracic epidural analgesia (TEA) [4]. There seems to be a shift from TEA to PVB when we compared our results to those reported in historical data, demonstrating that clinicians are willing to change their practice, when the available evidence is robust [18]. Other methods of regional anaesthesia used in thoracic surgery are muscular plane blocks (such as serratus anterior plane block and erector spinae block) and selective nerve blocks (such as pectoralis nerve and intercostal nerve block). Although the anatomy of these planes has been well known, the use of ultrasound has made visualizing the muscular plane/nerves much easier, thus reducing the rates of complications and increasing the success rate of the nerve block [19]. Intrapleural local anaesthesia, infiltration of local anaesthetic in the surgical incision, intercostal and subcostal drainage tube insertion sites are also useful in cardiothoracic surgery [20]. As the use of epidural is decreasing, it is likely that HCPs use a combination of other methods of regional anaesthesia such as paravertebral block and weak/strong opioids. This could partly explain why so many respondents report they use strong opioids. Another explanation would be the lack of resources, as in order to deliver multimodal anaesthesia, hospitals across the country need healthcare professionals that are trained in dealing with various nerve/muscle blocks. As several countries report they are battling an “opioid epidemic”, this aspect of ERAS warrant’s further research.

The compliance with the use of intercostal nerve sparring techniques when performing a thoracotomy was poor (23%). This can be partly explained by the fact that we surveyed various levels of providers, some of which might have an incomplete understanding of this aspect of patient care, hence a further survey of practice amongst surgeons might be warranted.

### Postoperative ERAS elements

Avoiding routine application of external suction and drain removal if the 24 h output is less than 420 ml, as long as serous fluid is drained are recommended [4]. The significant percentage of respondents who said they place drains on external suction reflects ‘traditional practice’, when drains were placed on -2 kPa of suction to promote pleural apposition and sealing of air leak. A 2015 meta-analysis on the benefits of applying suction in the post-operative period showed there is no improvement in clinical outcomes. Moreover, after conducting a national survey on chest drain management, the authors concluded that the clinical practice does not align with the level 1 evidence available, as 68% of participants reported they use suction [21]. Our findings demonstrate that the level of compliance has not changed dramatically, with 61% of respondents reporting they place the drains on suction. The free text analysis revealed a very important aspect: practice varies between consultants and most use the type of fluid drained (blood/chyle) or the presence of an air leak rather than the drain output to make a decision.

The importance of physiotherapy during postoperative period cannot be underestimated. Indeed, most responders follow the ERAS guidelines and do a routine physiotherapeutic assessment of all lobectomy patients (91%). A survey on physiotherapeutic provision in the UK reported similar results, with 97% of their respondents saying they routinely perform a physiotherapeutic assessment post-operatively [22]. Although studies do not show early mobilisation has any benefit in post-operative outcomes, we know that bed rest is harmful and the aim is to reduce the negative effects of immobilisation [23].

Due to the complexity of the interventions, high quality data supporting ERAS in patients undergoing lobectomy is lacking. A randomised control trial would be very challenging, but current literature supports further investigation, be it in the form of traditional clinical outcomes or by integrating patient reported outcomes or even implementation science [24]. By using a population based approach, one might be able to identify the high risk populations and gain more insight into how cost-effective an ERAS programme is. For example, a study by Mazza et al. [25] showed that adhering to ERAS has outcome benefits regardless of age or surgical approach and that ERAS adherence is a stronger predictor of length of stay than age. A study by Chen et al. [26] evaluating the application of ERAS to lung cancer patients showed outcomes benefits as well as improved nursing satisfaction when following ERAS principles. Furthermore, a study by Gonzales et al. [27], showed that an ERAS programme for VATS anatomical lung resection is not only cost effective, but also associated with a reduced length of stay and lower complications rate. This multitude of approaches demonstrates that ERAS contribution to outcomes if far more complex than current research is able to define. As compliance is related to the clinical effectiveness of ERAS [28], one has to ask the question: to what degree does compliance matter in an optimal ERAS protocol?

Overall, centres across the UK are actively developing their services to improve the ERAS programme implementation. A 2019 systematic review of qualitative studies exploring staff experiences of ERAS concluded that HCP have a positive attitude towards fast track surgery, but find the implementation process complex and challenging [29]. Evidence suggests that there is a gap between one’s perception and the “real” practice of ERAS [23], so we have chosen to survey individuals in an attempt to understand how practice differs across units due to subjective impressions. Furthermore, the uncertainty revolving around the respondent’s awareness of the ESTS-ERAS publications or it’s elements constitutes an important limitation of this survey. Hence, by publishing these results, we are likely to increase awareness and improve adherence to the ERAS guidelines.

## Conclusion

In conclusion, communication, teamwork and availability of resources are key elements to successfully implement ERAS. To improve adherence to ERAS, we propose dividing the tasks into easy to carry out remedial actions and more long term complex pathway development. For example, nutritional screening, fasting periods, chest drain management, postoperative analgesia and early mobilization are aspects that can be improved by raising awareness within individual units and are quick wins. Smoking cessation, prehabilitation, regional anaesthesia and surgical technique require a more complex intervention.

This national survey provides insight into “real life” ERAS practice in the UK and will be a driver for the other countries to undertake similar audits which will contribute to the development of an international ERAS implementation framework.

## Supplementary information

**Additional file 1.** Clinical care in patients undergoing lobectomy- A survey of Enhanced Recovery practice questionnaire.

## Data Availability

The datasets used and/or analysed during the current study are available from the corresponding author on reasonable request.

## Abbreviations

ERAS: Enhanced Recovery After Surgery
UK: United Kingdom
ESTS: European Society for Thoracic Surgery
VATS: Video-Assisted Thoracoscopic Surgery
HCP: Healthcare professional
DVD: Digital Versatile Disc
NSAIDs: Non-Steroidal Anti-Inflammatory Drugs
VTE: Venous Thrombembolism
NHS: National Health System
TEA: Thoracic Epidural Anaesthesia
PVB: Paravertebral Block
kPa: Kilopascal
GP: General Practitioner
NMDA: N-Methyl-D-aspartate
