# Supplementary material for: National survey of enhanced recovery after thoracic surgery practice in the United Kingdom and Ireland
Source: J Cardiothorac Surg. 2020 May 14;15:95. doi: 10.1186/s13019-020-01121-2 (PMC7227342; doi:10.1186/s13019-020-01121-2)
Supplement: Supplementary file 1 — Additional file 1. Clinical care in patients undergoing lobectomy- A survey of Enhanced Recovery practice questionnaire. [file 13019_2020_1121_MOESM1_ESM.docx]

**Supplementary material 1. Clinical care in patients undergoing lobectomy- A survey of Enhanced Recovery practice questionnaire**

1. **Do you work in a centre that performs lung lobectomy surgery?**

Yes

No

1. **Please select your centre**
2. **Please select your grade**

Consultant

Registrar

Senior nurse

Other

1. **In the preoperative setting, what form of counselling do your patients receive about their operation? (You may choose more than one option)**

Verbal information from doctor

Verbal information from specialist nurse

DVD

Website information (nhs choices/trust website)

Specific Information leaflet

Not sure

Other (please specify)

1. **Do you preoperatively screen patients for nutritional status?**

Yes

No

Unsure

1. **In patients who are identified as malnourished or at risk, what kind of support is offered? You may choose more than one option**

Dietary advice/Prescription for dietary supplements

Referral to dietician

Referral to GP

Not offered

N/A

Other (please specify)

1. **In patients who are current smokers, what support do you provide for smoking cessation? You may choose more than one option**

Nicotine replacement therapy prescribed in secondary care

Referral to hospital-based smoking cessation services

Referral to GP

Referral directly to community smoking cessation services

No routine support offered

Not sure

Other (please specify)

1. **What is the minimum amount of time you recommend to patients to abstain from smoking, ahead of surgery, in order to see benefits to their post-operative recovery?**

<2 weeks

>2 weeks to <4 weeks

>4weeks to <6 weeks

>6 weeks

Not sure

1. **In which group of patients do you offer pre-operative pulmonary rehabilitation (prehabilitation)?**

All patients

Only with Poor lung function/performance status

None

Other (please specify)

1. **Does your centre routinely start analgesic medication prior to surgery?**

Yes

No

Not sure

Other (please specify)

1. **Before surgery, how long are your patients kept nil by mouth of fluid?**

2 hours

>2 to <4 hours

>4 to <6 hours

>6hours

Other (please specify)

1. **Before surgery, how long are your patients kept nil by mouth of solids?**

<3 hours

>3 to <6 hours

6 hours

>6 hours

Other (please specify)

1. **Do your patients receive preoperative carbohydrate loading?**

Yes

No

Unsure

1. **When do your patients routinely receive VTE prophylaxis?**

Only whilst in hospital

Whilst in hospital and following discharge at home

Not sure

Other (please specify)

1. **Do your patients routinely receive a one off dose of antibiotic prior to surgical incision?**

Yes

No

1. **What does your centre routinely use as skin preparation solution**

Chlorhexidine

Poviodine

Not sure

Other (please specify)

1. **What method do you use for intraoperative warming? You may choose more than one option**

Forced air warming blankets

Heat mattress under the patient

Circulating water garments

Blankets

None

Not sure

Other (please specify)

1. **Where are your lobectomy patients routinely managed after surgery?**

Intensive Care Unit

General HDU

Thoracic HDU

Surgical ward

Other (please specify)

1. **What modalities does your centre routinely prescribe for post-operative nausea? You may choose more than one option**

5-hydroxytriptamine (5-HT3) receptor antagonists (e.g. ondansetron)

Dopamine agonists (metoclopramide/prochlorperazine)

Corticosteroids (e.g. dexamethasone)

Antihistamines (cyclizine)

Benzodiazepines (e.g. diazepam)

Anticholinergics (e.g. hyoscine)

Not sure

None

1. **What percentage of patients undergoing a thoracotomy will receive a thoracic epidural in your unit?**

<5%

6-25%

26-75%

76-100%

Not sure

1. **Which one of the following analgesic options do your patients receive post operatively? You may choose more than one option**

Paracetamol

Non-Steriodal Anti-inflammatories (e.g ibuprofen)

Weak opiods (e.g. codeine)

Strong opiods (e.g. morphine)

Neuropathic agents (e.g. gabapentin)

Local anaesthetic agents (e.g. lidocaine patches/injections)

NMDA antagonists (e.g. ketamine)

1. **Do your patients routinely receive aperients post operatively?**

Yes

No

Not sure

1. **How long after lobectomy do you routinely allow oral intake of fluid?**

<2hrs

>2 to <4hrs

>4 to <6hrs

>6hrs

Not sure

1. **How does your centre routinely try to prevent atrial fibrillation in the per-operative period? You may choose more than one option**

Avoid withdrawal of beta blockers pre-operatively

Peri-operative correction of magnesium/calcium

Prophylactic commencement of diltiazem in high risk post operative patients

Prophylactic commencement of amiodarone in high risk post operative patients

None of the above

1. **At your unit, what percentage of lobectomies do you estimate are performed using Video Assisted (VATS) techniques?**

<25%

26-50%

51-75%

76-100%

Not sure

1. **When performing a thoracotomy, do you routinely?**

Spare the muscle (do not divide latissmus or serratus anterior)

Use techniques to preserve the intercostal nerve

N/a

Other (please specify)

1. **What value of suction are your lobectomy patients places on post-operatively?**

0 kPa (gravity)

- 0.5 kPa

- 1 kPa

-1.5 kPa

-2 kPa

- 2.5kPa

> -2.5kPa

1. **What cut of value for pleural fluid drainage would you accept for removal of chest drain in the 1^st^ 24hrs?(free text)**
2. **Does your centre routinely catheterise (urinary) patients for perioperative monitoring?**

Yes

No

Not sure

1. **Which of your patients are routinely assessed after surgery by a physiotherapist?**

All lobectomy patients

Patients undergoing a Lobectomy via a thoracotomy

Only ‘high-risk’ patients

Not routinely assessed

1. **At your centre, which physiotherapy adjuncts are available in the management of patients following lobectomy? You may choose more than one option**

Incentive spirometry

Early mobilisation within 6 hours of surgery

Prophylactic mini-tracheostomy

Non-Invasive Positive Pressure ventilation

Not sure

Other (please specify)

1. **Do your patients routinely get discharged on opiate analgesia?**

Yes

No

Patients are not discharged with analgesia

Not sure

1. **Which post-operative concerns do your patients complain of most commonly? You may choose more than one option**

Pain

Uncertainty around diagnosis

Productive cough

Weakness

Nausea

Constipation

Mobility

1. **Are your patients provided with a point of contact for concerns on discharge?**

Yes

Not sure

1. **How soon after discharge will you follow-up patients?**

2 weeks

2-3 weeks

3-4 weeks

4-6 weeks

>6 weeks

**36 & 37. What aspects of ERAS are the most/least successful in your centre? You may chose more than one option**

Preadmission information/education/counselling

Availability of staff

Preoperative nutritional status

Smoking cessation

Alcohol cessation

Anaemia managements

Preoperative pulmonary rehabilitation programs

Preoperative fasting

Carbohydrate loading

Anaesthesia techniques(intubation/agents)

VTE prophylaxis

Asepsis (antibiotics/skin prep)

Intraoperative thermoregulation

Post oeprative ausea and vomiting control

Post-operative pain regimens

Fluid management

Atrial fibrillation prevention

Surgical technique/incision

Chest drain (inc management)

Urinary management

Physiotherapy

Discharged requirements

Follow-up

1. **Any other comments**
